# A patient and family data domain collection framework for identifying disparities in pediatrics: results from the pediatric health equity collaborative

**DOI:** 10.1186/s12887-018-0993-2

**Published:** 2018-01-31

**Authors:** Aswita Tan-McGrory, Caroline Bennett-AbuAyyash, Stephanie Gee, Kirk Dabney, John D. Cowden, Laura Williams, Sarah Rafton, Arie Nettles, Sonia Pagura, Laurens Holmes, Jane Goleman, LaVone Caldwell, James Page, Patricia Oceanic, Erika J. McMullen, Adriana Lopera, Sarah Beiter, Lenny López

**Affiliations:** 1 Massachusetts General Hospital, Disparities Solutions Center, 100 Cambridge Street, 16th floor, Boston, MA 02114 USA; 2 Mount Sinai Hospital, 600 University Avenue, Toronto, ON M4V 2C2 Canada; 3 Hospital for Sick Kids, 555 University Avenue, Toronto, ON M5G 1X8 Canada; 4 Nemours/Alfred I. duPont Hospital for Children, 1600 Rockland Rd, Wilmington, DE 19803 USA; 5 Children’s Mercy Hospital and Clinics, 2401 Gillham Rd, Kansas City, MO 64108 USA; 6 Holland Bloorview Kids Rehabilitation Hospital, 150 Kilgour Road, Toronto, ON M4G 1R8 Canada; 7 Seattle Children’s Hospital, 4800 Sand Point Way NE, Seattle, WA 98105 USA; 8 Monroe Carell Jr. Children’s Hospital at Vanderbilt, 2200 Children’s Way, Nashville, TN 37232 USA; 9 Nationwide Children’s Hospital, Childrens Dr ED-201, Columbus, OH 43205 USA; 10 Johns Hopkins Medicine, 1800 Orleans St, Baltimore, MD 21287 USA; 11 Cincinnati Children’s Hospital Medical Center, 3333 Burnet Ave, Cincinnati, OH 45229 USA; 12 University of California, San Francisco, 4150 Clement St, San Francisco, CA 94121 USA

**Keywords:** Pediatrics, Disparities, Race/ethnicity, Demographic data collection

## Abstract

**Background:**

By 2020, the child population is projected to have more racial and ethnic minorities make up the majority of the populations and health care organizations will need to have a system in place that collects accurate and reliable demographic data in order to monitor disparities. The goals of this group were to establish sample practices, approaches and lessons learned with regard to race, ethnicity, language, and other demographic data collection in pediatric care setting.

**Methods:**

A panel of 16 research and clinical professional experts working in 10 pediatric care delivery systems in the US and Canada convened twice in person for 3-day consensus development meetings and met multiple times via conference calls over a two year period. Current evidence on adult demographic data collection was systematically reviewed and unique aspects of data collection in the pediatric setting were outlined. Human centered design methods were utilized to facilitate theme development, facilitate constructive and innovative discussion, and generate consensus.

**Results:**

Group consensus determined six final data collection domains: 1) caregivers, 2) race and ethnicity, 3) language, 4) sexual orientation and gender identity, 5) disability, and 6) social determinants of health. For each domain, the group defined the domain, established a rational for collection, identified the unique challenges for data collection in a pediatric setting, and developed sample practices which are based on the experience of the members as a starting point to allow for customization unique to each health care organization. Several unique challenges in the pediatric setting across all domains include: data collection on caregivers, determining an age at which it is appropriate to collect data from the patient, collecting and updating data at multiple points across the lifespan, the limits of the electronic health record, and determining the purpose of the data collection before implementation.

**Conclusions:**

There is no single approach that will work for all organizations when collecting race, ethnicity, language and other social determinants of health data. Each organization will need to tailor their data collection based on the population they serve, the financial resources available, and the capacity of the electronic health record.

## Background

In the United States, the population, especially the pediatric population, is growing and projected to become more diverse. In 2011, the US Census Bureau reported that for the first time ever 50.4% of children in the US under the age of 1 were from minority groups [1]. The most recent US Census report of 2014 data indicated that the child population is projected to have more racial and ethnic minorities make up the majority of the population in 2020, and that by 2044, the US population would see this similar shift [2]. A report by the American Academy of Pediatrics (AAP) titled, “Race, Ethnicity, and Socioeconomic Status in Research on Child Health,” has found that disparities in pediatric care continues to be extensive, pervasive and persistent [3]. Disparities in pediatric health were noted across the spectrum of health and health care, including mortality rates, access to care and use of services, prevention and population health, chronic diseases, special health care needs, quality of care, and organ transplantation.

These disparities are likely to increase with the projected growth of children from minority groups in the US. Health care organizations will need to collect accurate and reliable data and stratify them by race, ethnicity, language and other social determinants of health in order to develop interventions to address disparities. This will also need to include the less explored frontiers of collecting data on sexual orientation, gender identity and disability. The Institute of Medicine (IOM) report *The Health of Lesbian, Gay, Bisexual, and Transgender People* recommends that data on sexual orientation and gender identity should be collected in the electronic health records (EHR), and most recently the Office of National Coordinator of Health Information Technology requires EHR systems certified under Stage 3 of Meaningful Use to allow users to collect data on sexual orientation and gender identity [4–6]. The IOM report on the Future of Disability in America recommends the creation of a comprehensive disability monitoring system, and the World Health Organization’s International Classification of Functioning, Disability and Health (ICF) provides a framework for measuring disability that has been endorsed by all WHO member states [7, 8]. Without accurate and reliable data collection we will not be able to understand nor address the root causes of disparities. The Affordable Care Act (ACA) underscores the importance of data collection through its section 4302, which requires the Secretary of Health and Human Services to establish data collection standards for race, ethnicity, sex, primary language, and disability for its programs and surveys that use self-reported data [9]. Collecting this data in a standardized fashion will help researchers better understand the impact of health care reform on reducing disparities while at the same time bolster efforts to monitor disparities. The AAP made a strong recommendation to prioritize research that understands and addresses disparities related to race, ethnicity and socioeconomic status, given that early life experience shape later life health outcomes [10].

Despite the aforementioned recommendations and legislation, the biggest challenge facing health care organizations is how to *operationalize* the data collection of race, ethnicity, language and other social determinants of health in a pediatric setting. The Health Research and Educational Trust (HRET) Disparities Toolkit provides national standards and guidance on data collection but nothing is specific to pediatrics [11]. The IOM report *Race, Ethnicity, and Language Data: Standardization for Health Care Quality Improvement* provides no guidance on what the unique operational challenges of collecting this data are in a pediatric setting, or how to collect it [12]. On a broader scope, there are no international criteria for data collection [13]. In sum, there is a dearth of best practices or standards in the US on how to best collect race and ethnicity data in a pediatric setting. In order to address these lacuna, a group of 16 research and clinical experts representing 10 pediatric care delivery systems in the US and Canada, formed the Pediatric Health Equity Collaborative (PHEC), with the goal of establishing sample practices, approaches and lessons learned with regard to race, ethnicity, language, and other demographic data collection in pediatric care settings, based on each institution’s experience and the demographic of the population they serve.

## Methods

### Formation of the pediatric health equity collaborative (PHEC)

In 2013, PHEC was formed and consisted of 16 research and clinical professional experts working in 10 pediatric care delivery systems in the US and Canada (8 pediatric and 2 pediatric/adult hospitals). This group convened twice in person for 3-day consensus development meetings and met multiple times via conference calls over a two year period. It is recommended that expert panels be multidisciplinary and inclusive of individuals from geographically diverse and culturally disparate areas allowing for breadth of experience and perspectives [14]. The panel consisted of researchers, pediatric clinicians, social workers, and diversity officers with expertise in pediatric healthcare disparities, quality improvement and performance measurement, and organizational change. Hospitals are located in Toronto, Canada and the following U.S. states: Delaware, Maryland, Massachusetts, Missouri, Ohio, Pennsylvania, Tennessee and Washington. These hospitals were a self-selected group of pediatric hospitals, the majority of who had participated in the Disparities Leadership Program (DLP) and were all focused on implementation of demographic data collection at their organization. The DLP is a year-long executive education program designed and developed by the Disparities Solutions Center at Massachusetts General Hospital for leaders in health care who want to address disparities by improving quality. This program has 3 aims: 1) to arm healthcare leaders with an understanding of the root cause of disparities and the vision to implement solutions and transform their organizations; 2) to help create a strategic plan or advance a project that reduces disparities; and 3) to align the goals of health equity with healthcare reform and value-based purchasing (https://mghdisparitiessolutions.org/the-dlp/). Given that the program focuses on implementation of solutions and leveraging the peer network of resources, this collaborative is a natural outgrowth and next step after the program. Three hospitals were from Toronto, Canada, while the remaining were US hospitals.

### Theme and content development

Current evidence on adult demographic data collection was systematically reviewed and unique aspects of data collection in the pediatric setting were outlined. Human centered design methods, developed by the Luma Institute, were utilized to facilitate theme development, facilitate constructive and innovative discussion, and generate consensus. All in-person and remote telephonic meetings were designed to facilitate open group discussion sessions which allowed participants to discuss and debate existing evidence; consider barriers to implementation and factors influencing local appropriateness; propose and clarify recommendations; and identify their logic and importance [15–17]. Human centered design techniques used included: Abstraction laddering (assists in defining a problem statement), Rose-Thorn-Bud (identifies issues and insights), affinity clustering (draws insights, new ideas, and patterns out of otherwise disparate pieces of information), Importance/Difficulty Matrix (prioritizes and develops a plan of action), Concept Poster (provides a road map for moving forward, and promotes a vision for the future) and Bull’s Eye Diagramming (ranks items in order of importance and sets priorities). In an iterative fashion, broad categories were narrowed, and consensus was reached on key themes and priorities for the paper. This iterative process was conducted over a three-day meeting of all participants in 2013. Concept mapping diagrams were developed, illustrated in poster form and photographed. All discussions were audio recorded for detailed theme analysis via content analysis by the group. The group refined the selection of data domains and conducted background research on data collection domains through a series of conference calls throughout the course of the year. The group selected 6 final domains; caregivers’ demographic data, race and ethnicity, language, sexual orientation and gender identity, disability, and social determinants of health. Each domain was assigned to a small working group who defined the domain, rationale for data collection, specified the data collection challenges for this data in a pediatric setting, and finally developed sample practices based on the group’s institutional experiences. In 2014, the group met a second time in person to finalize the discussion of the sample practices. After the second conference, all domain content was reviewed as a group through conference calls and electronically with all PHEC members.

## Results

We present the results below of each domain in the following format: context of the domain, rational for inclusion, challenges of collecting the domain data in a pediatric setting and sample practices.

### Caregiver considerations

#### Context

North American families are becoming more diverse and, as such, assumptions made about who the child’s primary caregiver is at healthcare appointments can lead to inconsistencies in data collection. For this reason, having a clear definition and scope for caregiver data collection is integral for the ability to understand how health outcomes in children may be impacted by their caregivers social determinants of health. Some organizations offer broad classification systems (e.g. including grandparents, roommates, etc.) while others use more narrow categories. For the purposes of data collection, identifying caregivers as the ‘main provider of economic and social support for a child or youth’ enables accurate comparisons and stratification of health outcome data.

#### Rationale for collecting data on the caregiver

A patient and family-centered approach to care recognizes the vital role of family in supporting the health and well-being of children and is responsive to the needs and preferences of patients, as well as their families [18]. Collecting demographic information from caregivers can assist healthcare providers in delivering care that meets the unique needs of children and their families, while being foundational for system level planning. Research demonstrates that a child’s health status is integrally associated with their family’s access to resources (e.g. income, housing, education), and thus caregiver demographics can also provide insight into the social, cultural, and economic factors that shape children’s health [19, 20].

#### Challenges in a pediatric setting

Several challenges exist when attempting to collect demographic data from caregivers. A primary challenge relates to the universal definition of the age of consent process for treatment and care decisions. There is no consistent approach based on using age versus capacity for decisions. Organizations are left to determine an age at which to move from administering surveys to caregivers to administering the survey to youth. This makes analysis of information across systems and locales more difficult. Challenges also exist with respect to capacity and determining appropriateness and ability for youth to complete the survey when developmental delay or cognitive impairment is present.

Other challenges include a lack of a formal policy on collecting patient demographics resulting in an inconsistent process, which may engender threats to data validity and risks to patient privacy. Furthermore, fear by youth that caregivers may access sensitive information (e.g. gender identity or sexual orientation questions) may lead to inaccurate response rates. Similarly, caregivers may be reluctant to provide information (e.g. income) that they do not want their child, other health care providers, or funding agencies to have access to. Organizations may face challenges with respect to response rates if caregivers or youth are not provided with a clear rationale for the purpose of data collection or do not feel privacy is adequately addressed.

The scope of what demographic data is collected must also be determined. While collecting a vast array of data will provide a more detailed landscape of caregiver and patient demographics, this practice is also highly resource intensive for organizations to collect, store and analyze and may not be supported by the electronic health record infrastructure.

#### Sample practices

Embedding privacy protocols into the collection, storage, and access to caregiver and patient demographic information will enhance the accuracy of reporting. If caregiver information is stored in the child’s health record, there will be a need for clear protocols around employee access to this information (including rationale for access), and transparency to the caregiver for meeting privacy regulations. As well, clearly defining the age at which youth will be asked demographic data is recommended prior to surveying this population while also sharing who may access this information. For example, Hospital for Sick Kids and Holland Bloorview Kids Rehabilitation Hospital in Toronto, Canada, have implemented a policy that children who are 13 and older respond to all demographic questions, except for income which is collected from the caregiver, and these hospitals do not collect data on sexual orientation or gender identity from patients who are 12 or younger.

Collecting caregiver data along with several similar child/youth based questions supports a more detailed understanding of the family’s demographics. To be meaningful, however, this data must align with the ability of the organization to analyze and use this data. A number of strategies can be employed to prioritize which demographic variables to collect and from whom. For example, variable selection may be health care driven. Demographics that are directly related to the provision of care (e.g. interpretation, religious affiliation, decision-aids) may be prioritized to advance current care practices.

### Race and ethnicity

#### Context

Race and ethnicity are concepts used to categorize large groups of people based on common origin or descent. Historically, race has related to physical characteristics and been assumed to have a biological basis, while ethnicity has related to culture or nationality. In recent decades, anthropological, genetic, and social research has cast doubt on a biological basis of race, leading to considerable overlap in current definitions of race and ethnicity [21, 22]. Both are now widely seen as dynamic, socially constructed categories of identity that change over time depending on political and historical context. Race and ethnicity are perceived identities (by the self and by the other), as opposed to objectively measurable characteristics. As a result, labeling varies with the labeler – a person’s own sense of race or ethnicity may be different from what an observer would assign them. Available labels also change. Historically, the options for racial labeling in the US have been determined by the government, especially through the census, with a broad array of changing terms used over the decades. In other countries, race may be seen differently or may have less prominence in governmental or other labeling systems. The dynamic nature of race and ethnicity harms their reliability and validity as data, challenging data collectors and analysts.

#### Rationale for collecting race and ethnicity data

Despite these challenges, the collection of data on patient race and ethnicity has been valuable in health care settings for multiple reasons: 1) race and ethnicity have been independently linked to disparate health and health care outcomes [3, 23, 24], 2) improving quality and safety of care for individuals (clinical care) and groups (public or community health) depends on understanding patient populations, 3) patient-provider racial and ethnic concordance can influence experiences and outcomes [25], and 4) reporting requirements often include race and ethnicity (e.g. research, funding, government programs). For many, race and ethnicity are important parts of personal and cultural identity, as well as determinants of individuals’ experiences in society at large. Health care providers and organizations can monitor and improve outcomes, as well as engage more effectively with patients and communities, when they know the racial and ethnic identity of those they serve.

#### Challenges in a pediatric setting

In concept and practice, racial and ethnic labeling presents multiple challenges in the pediatric setting:

1. *What labels do we use?-* Labels for self-identity change with time and differ by generation. Younger members of society can have different concepts of race than their caregivers, many seeing themselves as multiracial. How do we account for these changes in the pediatric setting?
2. *Whom are we labeling?-* Provider-caregiver interactions often matter as much as provider-patient interactions. Do we collect race/ethnicity of the caregiver, or only the child?
3. *Who is the labeler? -* Do caregiver and children share the same idea of what race/ethnicity the child is? If not, whose idea is right, and whose do we collect? Is there an age at which the child’s response takes precedence? Do two caregivers share the same idea of their child’s race?

#### Sample practices

In the US, existing recommendations made by the IOM [12] and the HRET [11] include categories for Hispanic ethnicity, race, and granular ethnicity (e.g. German, Kenyan, or Russian), with guidance on how to consider data options depending on local demographics and how they will be used. In Canada, race is not collected routinely, but ethnicity, visible minority status, and aboriginal identity may be included in governmental data systems [26]. In neither country is there guidance for collection of race, ethnicity, or related data in pediatric settings. Though we recommend that the existing basic standards (e.g. IOM and HRET in the US) be applied to pediatric settings, they are incomplete. To address the challenges described above, we offer the following pediatric considerations:

1. **Include “multiracial” and “multiethnic” as options, including the specific races or ethnicities (e.g. “black, white” or “German, American”).**
  Children identified in the US Census as having two or more races are increasing at a faster rate than in any single racial group [27]. Pediatric data systems must be prepared to accurately record their patients’ identities, as this changing demographic threatens the usefulness of traditional labeling systems.
2. **Collect race/ethnicity of caregivers.**
  Interactions with family members (particularly caregivers) are fundamental to effective pediatric care. Recording only child race/ethnicity ignores this fact, giving an incomplete picture of those being served.
3. **Collect the patient’s race from the patient.**
  Children’s sense of race/ethnicity develops over time and contributes significantly to their experience of family, peers, and others in society. Including it in the record starting at an appropriate age might allow pediatric providers and organizations to more completely understand their patients.

### Language

#### Context

As defined by the U.S. Department of Health & Human Services, individuals with Limited English Proficiency (LEP) are unable to communicate effectively in English because their primary language is not English and they have not developed fluency in the English language. Individuals should self-report their language preferences to ensure effective communication. Health care communication is complex in nature and requires comprehensive understanding [28].

#### Rationale for collecting data on language

Patients with LEP and their families are at a higher risk for miscommunication and less than optimal care [29–31]. The adverse events due to these risks have been documented in highly publicized legal cases leading to severe harm and even death [32]. Provider-patient language discordance is increasing due to the diversity of populations in the U.S. Language data collection is necessary to identify language needs, provide a professional medical interpreter and analyze health equity. In addition, language data collection ensures compliance with institutional and federal policies such as the Office of Minority Health’s National Standards for Culturally and Linguistically Appropriate Services (CLAS) in Health and Health Care and The Joint Commission’s 2015 Standards for the Hospital Accreditation Program. Literature has documented language collection best practices as asking patients [11]:

1. How well do you speak English?

- ○ Very well, well, not well, not at all, declined, unavailable

2. What is your preferred spoken language for care?
3. What is your preferred written language?
4. Do you need an interpreter?

- ○ Yes, no, don’t know, declined, unavailable

#### Challenges in a pediatric setting

In pediatrics, these questions should be asked of the child and of the care giver. Language discordance can occur (1) between provider-caregiver, (2) within caregivers (3) between child-caregiver and (4) between provider-child. In order to provide high quality pediatric care, effective communication with caregivers is essential. Over the years, the U.S. has implemented policies to provide language assistance to individuals with LEP [33–36]. These policies have been a catalyst to using professional medical interpreters, and not asking children to serve as interpreters for their guardians. Applying best practices in language collection to a pediatric setting would require asking the four above-mentioned questions of the patient and caregivers involved in the child’s care. However, for many health care systems collecting potentially 12 unique language elements for a pediatric family may be overly-complex and impractical given the number of questions, limited staff, and the capacity of the electronic health record infrastructure.

#### Sample practices

At a minimum, pediatric organizations should collect (1) Caregiver One’s preferred spoken language. Ideally, language data collection should also include (2) Caregiver One’s preferred written language, (3) Caregiver Two’s preferred spoken language, (4) Caregiver Two’s written spoken language (5) Patient’s preferred spoken language, and (6) Patient’s preferred written language (see Table 1).

**Table 1:** Sample Practice: Minimum language data collection

Language Domain	Patient	Caregiver 1	Caregiver 2
Preferred Spoken Language	English	English	Spanish
Preferred Written Language	English	Spanish	Spanish

### Caregiver’s preferred spoken language

Baseline data collection should include the preferred spoken language of a primary caregiver. If this is the only language field used, it should capture the language of the caregiver with limited English Proficiency. For example, if one caregiver is English proficient, and the other caregiver is not, this data element should capture the language of the caregiver with LEP. This prevents the other caregiver acting as an interpreter when they are both present, and also ensures that an interpreter is available at all the visits. Ideally, data should be collected on the preferred spoken language of a secondary caregiver, as there may be language discordance between the two caregivers.

### Caregiver’s preferred written language

It is important to remember that most of the patient’s care usually occurs outside of the clinical encounter. Therefore, assessing the preferred written language of the primary caregiver is essential to read and follow the instructions for medication administration, and recommendations regarding signs and symptoms to watch for, and when to return. Due to potential language discordance between caregivers, expanded language data collection should capture the preferred written language of two caregivers. Many IT systems do not include a choice of *does not read* within the preferred written language field. The assumption that the caregiver can read puts patient safety at risk.

### Patient preferred spoken and written language

As children develop they become active participants in their own health care. Therefore, collecting the patient’s preferred spoken and written language is relevant. Language discordance between the child and the caregiver is possible; for example, a deaf child of a hearing caregiver; an adopted child who speaks a different language than the caregiver; a bilingual child of monolingual caregiver or vice versa. This is commonly seen as children of immigrant caregivers become more fluent in English than their caregivers.

#### Other considerations

When designing your language collection electronic health record needs, determine the need for encounter-level data versus patient-level data. Encounter-level data is dynamic and can change from visit to visit. For example, depending on the caregiver that is accompanying the child to the appointment, a medical interpreter may or may not be needed. The responses within the preferred spoken and written language field should reflect the languages and dialects of the patient populations served. For example, Cincinnati Children’s LEP population includes Gulf Arabic, one of twenty-six Arabic dialects. The rapid development of language skills in children as well as the acquisition or loss of language skills in caregivers and children necessitates the revalidation of language data every two to three years.

### Gender identity and sexual orientation

Gender identity and sexual orientation are concepts that have become closely connected in research and advocacy. However, the IOM defines gender identity and sexual orientation as two separate terms [4]. As a result, ‘Definitions’ and ‘Rationale for Collection’ of gender identity and sexual orientation are discussed separately in this paper. However, the work on gender identity and sexual orientation was merged under ‘Pediatric Challenges’ and ‘Recommendations’ due to the many commonalities.

#### Context

##### Gender Identity

Gender identity is best defined as “a person’s basic sense of being a man or boy, a woman or girl, or another gender” [4]. In the case of trans people, gender identity does not reflect (biological) sex assigned at birth. Biological sex is birth-assigned and refers to the objectively measurable organs, hormones, and chromosomes. Gender identity therefore reflects a sense of “who I feel I am” while sex is a biological descriptor. Emerging research has debunked the assumption that children and youth who select gender nonconforming identities are ‘confused’; on the contrary, they show clear and consistent gender identities at both explicit and implicit levels [37].

A discussion on the collection of gender identity data should address the current issue of over-reliance on the collection of biological sex as a proxy/substitute for gender. Sex is limited to male, female, and the occasional inclusion of intersex. As explained above, gender identity is intended to go beyond biology by capturing a person’s subjective experience of who they are: male, female, gender queer, 2-spirit, etc., and is independent of biological sex. The use of sex as a proxy for gender identity is problematic for many reasons, including the propagation of gender binary, which is “the classification of sex and gender into 2 distinct and disconnected states of masculine and feminine”. It also maintains the exclusion of gender non-conforming persons, poses risks to provision of appropriate care, and perpetuates discrimination.

#### Context

##### Sexual Orientation

While gender identity is about the internal sense of the person as boy, girl, gender queer, etc., sexual orientation is used to express a person’s enduring emotional, romantic, and/or sexual attraction to another person(s) [38]. Though generally discussed in terms of exclusive categories (e.g. “gay”, “straight”), sexual orientation ranges along a continuum and may shift along a person’s life span. It is also important to note that sexual orientation does not define or determine sexual behavior (or activity), particularly among youth [39]; i.e. these two terms are not proxies for each other. It is critical to differentiate sexual orientation from other constructs such as behavior/activity when planning for both its collection and its use since they have different implications for clinical decisions and for assessing health disparities.

#### Rationale for collecting data on gender identity and sexual orientation

##### Gender Identity

The case for the collection of this data is a compelling one, from both a broader health disparities lens and from a clinical care perspective. Medical tests, growth charts, and laboratory results are primarily normed to biological sex. Therefore, access to information about biological sex, anatomy, and gender identity is often relevant to the provision of safe and appropriate health care, particularly for transgender patients. For example, transgender men are less likely to be current on Pap tests than non-transgender women, despite the fact that transgender men may retain their natal reproductive organs [40]. In comparison to persons who conform to sex-based social expectations, persons with non-conforming gender identities are significantly more likely to experience social and family violence, homelessness, harassment, bullying, and blatant discrimination [41, 42]. Children and youth are particularly susceptible to bullying, with one statistic indicating that 78% of trans K-12 are targeted by bullies [42]. As a result, adolescents with gender nonconforming identities exhibit higher rates of high-risk behavior [43] and adverse mental health outcomes including post-traumatic stress disorder, depression, suicidal ideations, and anxiety [44, 45].

Addressing the negative impact of these adverse conditions on health, coping, and arising needs is essential for the provision of effective health care for adolescents*.* That may include having a conversation on the stressors and challenges that a patient is dealing with and providing health care support or interventions as needed. Ways that health care organizations use this information to inform practice include identifying patients’ preferred name and pronoun, providing access to gender neutral washrooms, and assigning rooms to ensure patient safety.

While the adverse outcomes experienced by gender non-conforming youth have been well-established, the scarcity of evidence-based and tested data collection efforts pose a major challenge to understanding and reducing these disparities.

##### Sexual Orientation

The wide range of disparities for children and youth identifying as lesbian, asexual, gay, bisexual, 2-spirit, queer, questioning, and other sexual dimensions include higher rates of suicidal ideations [46], emotional distress [47], increased risky behavior (e.g. misuse of prescription drugs) [48], experiences of harassment and bullying [49], and disproportionate representation among homeless youth [50]. Patients seeking care are also often faced with heteronormativity: the assumption that everyone is heterosexual. This assumption impacts clinical decisions and interactions, health care planning, development of best practices, and health research topics. The American Academy of Pediatricians also recognized the adverse impact of heteronormative practices and issued policy recommendations specifically targeting heterosexism [51]. Taken together, homophobia and heterosexism have been linked to adverse health outcomes, distrust of medical professionals, and avoidance of the medical system [52].

Efforts on the collection and use of data on sexual orientation continue to be dispersed. More importantly, available data sources are often not easily applied to health care research, increasing the need for health care driven efforts for collecting this data.

#### Challenges in the pediatric setting

A number of issues need to be addressed by health care organizations planning for patient demographic data collection on gender identity and sexual orientation. A primary consideration is the protection of this information and patient safety, particularly in pediatric settings. Since most interactions with pediatrics happen in the presence of a caregiver, collecting this information can trigger conversations that the patient has not yet had or is not ready to have. As highlighted earlier, the experience of those patients may include violence within the family, even expulsion from their home. Therefore, data collection methodologies should ensure protections and supports for children and youth who share this information.

A second issue concerns the fluidity of sexual orientation among youth and children, who may resist labels and label meanings [53]. Exploration of gender expression and identity is part of childhood development and is not necessarily constant throughout childhood and adolescence. [4]. In many cases children’s gender nonconforming behavior does not translate to gender nonconforming identities later on [54]. Developmental trajectories therefore pose a unique challenge to the collection of this data and highlight a need to acknowledge the fluidity of gender identity and sexual orientation among pediatric populations.

A third issue focuses around the logistics of collecting this data, particularly resistance from data collectors and their prevalent belief that patients under 18 should not be asked about issues relating to non-gender conforming behaviors/attitudes and sexual orientation. This is an issue that at least one of the hospitals on this paper has faced and may be more challenging in pediatrics than adult hospitals.

#### Sample practices

Starting or strengthening data collection in areas of gender identity and sexual orientation should consider the following:

1. **What is the purpose of collecting this information?** Defining the purpose will shape the question being used and strengthen its validity (e.g. orientation versus specific behaviors, biological sex versus gender identity, etc).
2. **Be aware of the fluidity of responses**, which can have implications for tracking data and understanding how needs and supports may be shaped by those experiences.
3. **Identify practices and policies that ensure patient privacy** when asking questions and saving responses. This may include consulting health records staff, social workers, or the legal department on how data is collected, stored, and disclosed.
4. **Address staff resistance to collecting this data through training** that clarifies concepts of gender identity and sexual orientation. This can raise awareness on existing disparities, and encourage staff to be allies to patients and their caregivers.

### Disability

#### Context

The definition of disability for the purpose of data collection was difficult to determine as social context influences this construct. The World Health Organization’s (WHO) International Classification of Impairments, Disabilities and Handicaps (1980) defines impairment as a loss of function, disability as the resultant restriction to activity and handicap as the disadvantage that limits participation [55]. These three areas all informed the Sample Practices for this domain.

#### Rationale for data collection on disability

Disabilities can have an impact on social exclusion, early childhood development and learning, as well as barriers to income earning through meaningful employment. Understanding health impacts through the disability lens acknowledges the factors that contribute to further marginalization. While caregivers with disabilities may have less access to income and experience societal challenges it is also true that pediatric disabilities impact the family as a whole. In order to collect disability data that is meaningful, organizations need to clearly define the rationale for their questions. Data collection for the purpose of advocacy for enhanced supports may look different than those questions that determine individualized care and treatment plans.

#### Challenges in the pediatric setting

The following challenges were identified in response to capturing disability demographic data:

1. *What labels do we use?*

Disability is rarely captured through a static diagnosis but, instead, presents as a social construct. What labels would sufficiently determine a reduction in activity due to disability?

2. *Whom are we labeling?*

Are childhood disability and caregiver disability both relevant for data collection purposes?

3. *Who is the labeler?*

Based on the WHO classification, disability would be captured through an identification of restricted activity and not clinical diagnosis. In this case, who defines this restriction?

#### Sample practices

Despite the difficulties and challenges with collecting this information, several opportunities were identified as sample practice recommendations.

1. **Look to the legislation for guidance.**
  Examples:

- In Ontario, Canada the Accessibility for Ontarians with Disabilities Act mandates how individuals must be accommodated by businesses and employers.
- In the United States, section 4302 of the Affordable Care Act mandates demographic data collection, including disability status.

Legislation and policy can be significant drivers in this process.

2. **Disability data should be based on symptomology and/or accommodations.** Disability data should be stratified with other demographic questions. Clinical diagnosis does not accurately reflect a level of impairment or participation in society. Individuals with disability may be more or less impacted when this data is stratified with income, education and other social supports.
3. **Collect disability data of caregiver(s) and children/youth**
  Caregivers with disabilities may experience barriers to social inclusion and income that can impact health outcomes for other family members. Childhood disability can reduce caregiver income and create barriers to participation, ultimately impacting health outcomes.
4. **Disability data should be collected frequently (at minimum, every 2–3 years)**
  Disability status can change over time, depending on the clinical diagnosis or other rehabilitation factors.

### Social determinants of health

#### Context

Data collection to help to identify health and healthcare disparities has traditionally included the collection of Race, Ethnicity and Language (REaL) data. While the collection of REaL data assists with the identification of disparities, it does not necessarily assist with understanding the major influencers of these disparities. Ideally, data collection would lead to a better understanding of the root causes of disparities within racial and ethnic groups and what strategies would provide more culturally competent care. This may be especially true within the pediatric population where the socio- cultural factors of more than one caregiver may determine the future health of the child, including the development of a future healthcare disparity. The IOM recommends collection of 11 core domains and 12 measures of social and behavioral factors in electronic health records (EHR) [5]. The final set of measures include: alcohol use, race and ethnicity, residential address, tobacco use and exposure, census tract-median income, depression, education, financial resource strain, intimate partner violence, physical activity, social connections and social isolation, and stress. Although it should be noted that these are 11 core domains, the IOM committee identified additional domains for *consideration* for inclusion an all EHRs (sexual orientation, country of origin, employment, health literacy, physiological assets, and dietary patterns).

#### Rationale for collecting data on social determinants of health

Collecting social determinants of health data is important for healthcare organizations to better understand the populations that they serve. Collecting this data in a standardized way in the electronic medical record allows for the improved efficiency of multiple caretakers viewing the same data without the need for individual providers replicating its collection during each separate encounter. Understanding the social context of a child’s family is imperative to understanding the social determinants of health of all communities and populations in order to better facilitate public preventative health interventions. In addition, the collection of social data on individual caregivers informs the provider about the social influences on each child’s health and potential barriers to their treatment.

#### Patient story

1. A 10 year old African American male with uncontrolled asthma was given a prescription for a nebulizer 10 months ago. The family was socioeconomically disadvantaged. After collecting data on social determinants of health, it was discovered that the family did not have consistent electricity, a requirement for the use of the nebulizer. After learning this piece of social data, the family was given assistance and the child’s asthma improved.

#### Challenges in the pediatric setting

Unique to the pediatric population is that the child may have multiple diverse caregivers, and may reside in more than one family structure, setting, and community. Accordingly, different cultural and social settings may influence that child’s health and healthcare. Collecting data on each of these caretakers and settings, and determining which measure should be asked of the patient versus the caregiver, or both may provide an even greater challenge.

#### Sample practices

The majority of domains suggested by the IOM report are not routinely collected in clinical settings. Because of the broad scope of these measures, what data to collect will be in large part determined by an organization’s capacity and resources, EHR system, populations served, and will vary by organization’s needs. For example, in order to avoid undue burden on the registration staff, one pediatric hospital participating in PHEC piloted the collection of this data through pad technology and the use of an EHR home portal accessible via home computer or smart phone application. Initial feedback from both clinicians and caretakers was positive. Future steps include creating provider alerts within the EHR to alert for potential cultural and social barriers to successful treatment of the child, along with links/triggers for social worker/care coordinator/patient navigator support of the child. Two other pediatric institutions in Canada participate in a citywide initiative to collect an extensive pediatric social/cultural data set, which includes religious or spiritual affiliation, sexual orientation, income and country of origin [56]. Current practices at various pediatric healthcare institutions are listed in the Pediatric Data Collection Domains and Sample Practices Table [57].

## Discussion

Group consensus determined six final data collection domains: 1) caregivers’ demographic data, 2) race and ethnicity, 3) language, 4) sexual orientation and gender identity, 5) disability, and 6) social determinants of health. For each domain, the group defined the domain, established a rational for collection, identified the unique challenges for data collection in a pediatric setting, and developed sample practices. The sample practices presented are based on the experience of the members of PHEC as a starting point to allow for customization unique to each health care organization. Health care organizations providing care to pediatric patients will have to consider the following when implementing data collection systems:

1. Health care organizations should determine the purpose of the data collection before they address the challenges of operationalizing the implementation of the data collection on these domains.
2. Given that the care of the patient extends beyond the patient to the family and the social environment in which the patient is raised, health care organizations should include data on the caregiver(s) of the patient.
3. Since there is no universal definition of the age of consent process for treatment and care decisions, health care organizations will have to determine an age at which it is appropriate to collect data from the patient instead of the caregiver. For example, in Toronto, hospital guidelines are to collect demographic information from patients who are 14 years and older. For patients who are 13 years and under, this information will be asked from a caregiver. The exception to this is the collection of data on sexual orientation and gender identity, which is only asked from patients who are 14 and older.
4. Given the changing nature of pediatrics and the life span it covers, it’s important to collect data on these domains not just once but multiple times since patient and caregiver preferences may change over the course of time.
5. Health care organizations may be limited by the capacity of their electronic health record in what information they would *like* to collect versus *what is feasible* operationally.

The ability of hospitals and other health care organizations to identify and address racial/ethnic disparities hinges on their collecting information about their patients’ race and ethnicity. This essential step was recommended in *Unequal Treatmen*t [24] and was emphasized by a group of twenty experts from the fields of racial/ethnic disparities in health care, quality improvement and organizational excellence who were convened by the Disparities Solutions Center in 2006 for a one-and-a-half-day Strategy Forum. This group of experts recommended race and ethnicity data collection as an integral foundation to address racial and ethnic disparities [58]. Quality improvement efforts to monitor for differences by non-clinically relevant characteristics such as demographic data are often hampered by the lack of detailed demographic data collection. There is evidence that hospitals can collect REaL data in a reliable fashion across multiple clinical care settings and successfully use the data in quality improvement and performance monitoring [59, 60].

### Limitations

Due to the lack of national and international guidelines for pediatric demographic data collection, practice guideline development relied on a consensus-based approach. The primary limitation of consensus development panels is the potential for bias in the recruitment of panel members thus leading to non-nationally representative practice suggestions. We attempted to minimize this bias by recruiting panel members from different US states and countries and a range of professional backgrounds, use of an experienced facilitator, and use of formal consensus development methods and structured group interaction to promote the involvement of all panel members. The development of these sample practices was based on an integrated program of critical evidence review and discussion. Finally, we present sample practices from a broad range of pediatric healthcare delivery systems and do not present formal detailed guidelines given the heterogeneity and complexities of individual health system data collection capacity and methodology. Our approach of presenting data collection domains follows prior US National Academy of Medicine guidelines for data collection which specified data domains and not detailed implementation methods maximizing healthcare delivery system tailoring to their own unique settings [12]. Thus, our work lays the foundation for future pediatric societies to engage in formal demographic data guideline development.

## Conclusion

By 2020, the child population is projected to be a majority-minority population [2] and health care organizations will need to have a system in place that collects accurate and reliable data in order to monitor disparities. A very thoughtful approach should be taken by organizations when considering what data to collect and for what purpose. There is not one approach that will work for all organizations when collecting race, ethnicity, language and other social determinants of health data. Similar to cultural competent care, each organization will need to tailor their data collection based on the population they serve, the financial resources available, and the capacity of the electronic health record.

With the changing demographics of the pediatrics populations in the US and around the world, health care organizations are seeking guidance on how best to collect the above data domains. PHEC has framed the development of these sample practices as *based on the experiences of the members of the group* with the hopes that this would provide a starting point for health care organizations collecting data to address root causes of disparities.

## Abbreviations

AAP: American Academy of Pediatrics
ACA: Affordable Care Act
CLAS: Culturally and Linguistically Appropriate Services
DLP: Disparities Leadership Program
EHR: Electronic health records
HRET: Health Research and Educational Trust
IOM: Institute of Medicine
LEP: Limited English Proficiency
PHEC: Pediatric Health Equity Collaborative
WHO: World Health Organization’s

## References

[1] US Census Bureau (2012). Most children younger than age 1 are minorities, Census Bureau reports.

[2] Colby SL, Ortman JM (2014). Projections of the size and compositions of the US population: 2014 to 2060, current population reports.

[3] Flores G, Committee On Pediatric Research (2010). Technical report--racial and ethnic disparities in the health and health care of children. Pediatrics.

[4] Institute of Medicine Committee on Lesbian G, Bisexual, and Transgender Health Issues and Research Gaps and Opportunities. The Health of Lesbian, Gay, Bisexual, and Transgender People. Building a Foundation for Better Understanding. Washington: National Academies Press; 2011. 22013611

[5] Committee on the Recommended Social and Behavioral Domains and Measures for Electronic Health Records; Board on Population Health and Public Health Practice IoM (2014). Capturing social and behavioral domains and measures in electronic health records: phase 2.

[6] Department of Health and Human Services Office of the Secretary (2015). 2015 edition health information technology (health IT) certification criteria, 2015 Edition Base electronic health record (EHR) definition, and ONC health IT certification program modifications.

[7] The Institute of Medicine Committee on Disability in America. The Future of Disability in America. Washington: National Academies Press; 2007.

[8] International Classification of Functioning, Disability and Health (ICF) [http://www.who.int/classifications/icf/en/].

[9] Dorsey R, Graham G, Glied S, Meyers D, Clancy C, Koh H (2014). Implementing health reform: improved data collection and the monitoring of health disparities. Annu Rev Public Health.

[10] Cheng TL, Goodman E, Committee on Pediatric R (2015). Race, ethnicity, and socioeconomic status in research on child health. Pediatrics.

[11] Hasnain-Wynia R, Pierce D, Haque A, Hedges Greising C, Prince V, Reiter J (2007). Health Research and Educational Trust Disparities Toolkit.

[12] Ulmer C, McFadden B, Nerenz DR, Institute of Medicine (U.S.) (2009). Subcommittee on standardized collection of race/ethnicity data for healthcare quality improvement board on health care services. Race, ethnicity, and language data: standardization for health care quality improvement.

[13] United Nations, Department of Economic and Social Affairs, Statistics Division (2014). Principles and recommendations for a vital statistics system, revision 3.

[14] Fretheim A, Schunemann HJ, Oxman AD (2006). Improving the use of research evidence in guideline development: 3. Group composition and consultation process. Health Res Policy Syst.

[15] Matheson GO, Pacione C, Shultz RK, Klugl M (2015). Leveraging human-centered design in chronic disease prevention. Am J Prev Med.

[16] Giacomin J (2014). What is human centred design?. Des J.

[17] Harvard Business Review. A Taxonomy of Innovation (Based on the work of Luma Institute). Harvard Business Review; 2014.

[18] Conway J, Johnson B, Edgman-Levitan S, Schlucter J, Ford D, Sodomka P, Simmons L (2006). Partnering with patients and families to design a patient- and family-centered health care system: a roadmap for the future- a work in progress.

[19] Case A, Lubotsky D, Paxson C. Economic status and health in childhood: The origins of the gradient. National Bureau of Economic Research; 2001. 10.1257/000282802762024520 29058397

[20] Mikkonen J, Raphael D (2010). Social determinants of health: the Canadian facts.

[21] Wade P (2007). Race, ethnicity and nation: perspectives from kinship and genetics.

[22] Oppenheimer GM (2001). Paradigm lost: race, ethnicity, and the search for a new population taxonomy. Am J Public Health.

[23] Centers for Disease Control and Prevention. Health Disparities and Inequalities Report-United States. MMWR. 2013;62(Suppl 3).

[24] Smedley BD, Stith AY, Nelson AR. Unequal Treatment: Confronting racial and ethnic disparities in health care (full printed version): National Academies Press; 2002. 25032386

[25] Cooper LP, NR. Disparities in Patient Experiences, Health Care Processes, and Outcomes: The Role of Patient-Provider Racial, Ethnic, and Language Concordance. The Commonwealth Fund; 2004.

[26] Thomas DM, Biette DN (2014). Canada and the United States: differences that count.

[27] Federal Interagency Forum on Child and Family Statistics (2014). Race and Hispanic origin composition: percentage of US children ages 0–17 by race and Hispanic origin, 1980–2013 and projected 2014–2050.

[28] Baker DW (2006). The meaning and the measure of health literacy. J Gen Intern Med.

[29] Karliner LS, Jacobs EA, Chen AH, Mutha S (2007). Do professional interpreters improve clinical care for patients with limited English proficiency? A systematic review of the literature. Health Serv Res.

[30] Schiaffino MK, Al-Amin M, Schumacher JR (2014). Predictors of language service availability in U.S. hospitals. Int J Health Policy Manag.

[31] Divi C, Koss RG, Schmaltz SP, Loeb JM (2007). Language proficiency and adverse events in US hospitals: a pilot study. Int J Qual Health Care.

[32] Johnstone MJ, Kanitsaki O (2006). Culture, language, and patient safety: making the link. Int J Qual Health Care.

[33] Chen AH, Youdelman MK, Brooks J (2007). The legal framework for language access in healthcare settings: title VI and beyond. J Gen Intern Med.

[34] Civil Rights Act of 1964. In: Title VI, 42 USC § 2000d et seq. United States.

[35] United States. Office of Minority Health. National Standards for Culturally and Linguistically Appropriate Services in Health Care. Washington: Office of Minority Health, US Department of Health and Human Services; 2001.

[36] Executive Order 13166 (2000). Improving access to services for persons with limited English proficiency.

[37] Olson KR, Key AC, Eaton NR (2015). Gender cognition in transgender children. Psychol Sci.

[38] Just the Facts Coalition (2008). Just the facts about sexual orientation and youth: a primer for principals, educators, and school personnel.

[39] Mustanski B, Kuper L, Greene G, Tolman D, Diamond L (2014). Development of sexual orientation and identity. Handbook of sexuality and psychology.

[40] Peitzmeier SM, Khullar K, Reisner SL, Potter J (2014). Pap test use is lower among female-to-male patients than non-transgender women. Am J Prev Med.

[41] Haas A, Rodgers P, Herman J. Suicide attempts among transgender and gender non-conforming adults: findings of the national transgender discrimination survey. In.: American Foundation for Suicide Prevention, Williams Institute; 2014.

[42] Grant J, Mottet L, Tanis J, Harrison J, Herman J. Injustice at every turn: a report of the national transgender discrimination survey. District of Columbia: National Center for Transgender Equality and National Gay and Lesbian Task Force; 2011.

[43] Reisner SL, Greytak EA, Parsons JT, Ybarra ML (2015). Gender minority social stress in adolescence: disparities in adolescent bullying and substance use by gender identity. J Sex Res.

[44] Reisner SL, Vetters R, Leclerc M, Zaslow S, Wolfrum S, Shumer D, Mimiaga MJ (2015). Mental health of transgender youth in care at an adolescent urban community health center: a matched retrospective cohort study. J Adolesc Health.

[45] Roberts AL, Rosario M, Corliss HL, Koenen KC, Austin SB (2012). Childhood gender nonconformity: a risk indicator for childhood abuse and posttraumatic stress in youth. Pediatrics.

[46] Ybarra ML, Mitchell KJ, Kosciw JG, Korchmaros JD (2015). Understanding linkages between bullying and suicidal ideation in a national sample of LGB and heterosexual youth in the United States. Prev Sci.

[47] Robinson JP, Espelage DL, Rivers I (2013). Developmental trends in peer victimization and emotional distress in LGB and heterosexual youth. Pediatrics.

[48] Kecojevic A, Wong CF, Schrager SM, Silva K, Bloom JJ, Iverson E, Lankenau SE (2012). Initiation into prescription drug misuse: differences between lesbian, gay, bisexual, transgender (LGBT) and heterosexual high-risk young adults in Los Angeles and New York. Addict Behav.

[49] Kosciw JG, Greytak E, Palmer N, Boesen M. The 2013 National School Climate Survey: the experiences of lesbian, gay, bisexual and transgender youth in our nation’s schools. New York: GLSEN. p. 2014.

[50] Corliss HL, Goodenow CS, Nichols L, Austin SB (2011). High burden of homelessness among sexual-minority adolescents: findings from a representative Massachusetts high school sample. Am J Public Health.

[51] Levine DA, Committee on Adolescence (2013). Office-based Care for Lesbian, gay, bisexual, transgender, and questioning youth. Pediatrics.

[52] Fish J (2006). Heterosexism in health and social care.

[53] Coleman-Fountain E (2014). Lesbian and gay youth and the question of labels. Sexualities.

[54] Steensma TD, McGuire JK, Kreukels BP, Beekman AJ, Cohen-Kettenis PT (2013). Factors associated with desistence and persistence of childhood gender dysphoria: a quantitative follow-up study. J Am Acad Child Adolesc Psychiatry.

[55] World Health Organization (1980). International classification of impairments, disabilities, and handicaps: a manual of classification relating to the consequences of disease, published in accordance with resolution WHA29. 35 of the twenty-ninth world health assembly, may 1976.

[56] Wray R, Agic B, Bennett-AbuAyyash C, Kanee M, Lam R, Mohamed A, Tuck A (2013). We ask because we care: the tri-Hospital and TPH health equity data collection research project report September 2013.

[57] Pediatric Data Collection Domains and Sample Practices [https://mghdisparitiessolutions.files.wordpress.com/2015/10/phec_datacollectiondomainssamplepracticesaugust2015.pdf].

[58] King RK, Green AR, Tan-McGrory A, Donahue EJ, Kimbrough-Sugick J, Betancourt JR (2008). A plan for action: key perspectives from the racial/ethnic disparities strategy forum. Milbank Q.

[59] Siegel B, Sears V, Bretsch JK, Wilson M, Jones KC, Mead H, Hasnain-Wynia R, Ayala RK, Bhalla R, Cornue CM (2012). A quality improvement framework for equity in cardiovascular care: results of a national collaborative. J Healthc Qual.

[60] Bhalla R, Yongue BG, Currie BP (2012). Standardizing race, ethnicity, and preferred language data collection in hospital information systems: results and implications for healthcare delivery and policy. J Healthc Qual.

